# Seminal Plasma: Relevant for Fertility?

**DOI:** 10.3390/ijms22094368

**Published:** 2021-04-22

**Authors:** Heriberto Rodriguez-Martinez, Emilio A. Martinez, Juan J. Calvete, Fernando J. Peña Vega, Jordi Roca

**Affiliations:** 1 Department of Biomedical & Clinical Sciences (BKV), BKH/Obstetrics & Gynaecology, Faculty of Medicine and Health Sciences, Linköping University, SE-58185 Linköping, Sweden; 2 Department of Medicine and Animal Surgery, Faculty of Veterinary Medicine, International Excellence Campus for Higher Education and Research “Campus Mare Nostrum”, University of Murcia, 30100 Murcia, Spain; emilio@um.es (E.A.M.); roca@um.es (J.R.); 3 Laboratorio de Venómica Estructural y Funcional, Instituto de Biomedicina de Valencia, C.S.I.C., 46010 Valencia, Spain; jcalvete@ibv.csic.es; 4 Laboratory of Equine Reproduction and Equine Spermatology, Veterinary Teaching Hospital, 10003 Caceres, Spain; fjuanpvega@unex.es

**Keywords:** epididymis, accessory sexual glands, ejaculate, seminal fluid, proteome, cytokines, antioxidants, gene expression, female genital tract, fertility, livestock, human

## Abstract

Seminal plasma (SP), the non-cellular component of semen, is a heterogeneous composite fluid built by secretions of the testis, the epididymis and the accessory sexual glands. Its composition, despite species-specific anatomical peculiarities, consistently contains inorganic ions, specific hormones, proteins and peptides, including cytokines and enzymes, cholesterol, DNA and RNA—the latter often protected within epididymis- or prostate-derived extracellular vesicles. It is beyond question that the SP participates in diverse aspects of sperm function pre-fertilization events. The SP also interacts with the various compartments of the tubular genital tract, triggering changes in gene function that prepares for an eventual successful pregnancy; thus, it ultimately modulates fertility. Despite these concepts, it is imperative to remember that SP-free spermatozoa (epididymal or washed ejaculated) are still fertile, so this review shall focus on the differences between the in vivo roles of the SP following semen deposition in the female and those regarding additions of SP on spermatozoa handled for artificial reproduction, including cryopreservation, from artificial insemination to in vitro fertilization. This review attempts, including our own results on model animal species, to critically summarize the current knowledge of the reproductive roles played by SP components, particularly in our own species, which is increasingly affected by infertility. The ultimate goal is to reconcile the delicate balance between the SP molecular concentration and their concerted effects after temporal exposure in vivo. We aim to appraise the functions of the SP components, their relevance as diagnostic biomarkers and their value as eventual additives to refine reproductive strategies, including biotechnologies, in livestock models and humans.

## 1. Introduction

What is fertility among species with internal fertilization? Fertility is their capacity (males and females) to produce offspring, either born from an egg or after carrying a pregnancy to term. In avian species fecundity, e.g., the potential for fertility is the most relevant in terms of gamete production and fertilization. In mammals, and particularly in eutherian species, fertility is the crucial capacity of bearing a partially hemi-allogeneic individual (the fetus) or parts of a decisive organ as the placenta. Some species are polytocous adding another level to fertility: prolificacy, imposing a grading of the capacity to fertilize and carry to term litters of various sizes.

How does semen deposition in the female influence fertility? Semen entry in the female genital tract is a pre-requisite for internal fertilization, with spermatozoa still being the most relevant factor for fecundity in terms of numbers and normality of function. However, is there any other factor than spermatozoa affecting fertility? Yes, the seminal plasma (SP), because it modulates sperm viability and function but also affects the ability of spermatozoa to interact with the lining epithelium of the female genital tract and its secretions; it even serves as a carrier of signals for the female, their immune system in particular [1,2,3,4]. However, these roles are considered not essential for fertilization, as proved by the use of epididymal or of washed ejaculated spermatozoa for in vitro fertilization (IVF), or intra-cytoplasmatic sperm injection (ICSI) and artificial reproductive techniques (ARTs) of increasing application to alleviate the increasing infertility seen in humans, thought to be related to the detrimental impacts of the environment and certain habits on male health [5,6,7]. Under the procedures of IVF and/or sperm cryopreservation, the SP has even been considered detrimental and for decades customarily removed [8,9]. However, SP seems to warrant functions beyond fertilization, and is therefore related—in many species—with fertility [5,10,11,12,13]. Noteworthy, human SP has not been explored for eventual accompanying deterioration as what spermatozoa had been [6]. In practice, and often as a consequence of empirical experiments, a minimal proportion of SP has been left present when semen is processed to produce a semen dose for artificial insemination (AI), in order to maintain a fertility similar to what is seen after natural mating [14,15,16,17,18,19,20,21,22,23]. However, the intrinsic reasons for what is present in those SP proportions remains unknown.

Seminal plasma: what is it, and what roles does it play? The SP is a composite fluid built by the concerted mixture of secretions from the testis, epididymis and the accessory sexual glands, with distinct species differences that, together with emitted aliquots of the epidydimal sperm reserves, compose the ejaculate [24]. The SP plays several roles, including modulation of sperm function, of their ability to generate energy using substrates present in the SP, to interact with the epithelia and the secretions of the female genital tract and also as a carrier of signals for the female, for their immune system in particular. The latter implies the female combats the entry of pathogens and of cells and proteins/peptides immunologically foreign to the female by eliciting a transient genital inflammation [25]. However, considering spermatozoa are also foreign cells, yet essential for fertilization, such transient inflammation is followed by the establishment of a longer-lasting state of immunological tolerance towards those spermatozoa that fortuitously colonize the oviductal sperm reservoirs [11,26,27].

Is it just the SP proteome? The SP proteome appears to participate in this double signaling, triggering a transient inflammation and the changes in the expression of genes related to immune processes at the female internal genitalia [28,29,30]. The latter transcriptomic events ultimately modulate sperm rejection or tolerance, perhaps even disclosing the relative intrinsic fertility of the male and/or female. The proteome and peptidome of semen, including proteins and peptides derived from spermatozoa and the various fractions of the SP the gametes bathe in, has been intensely studied over the past decade, with particular attention to the structure, temporal distribution and function of the major proteins [31,32,33,34,35,36]. As well, transcriptomic studies have signaled that many genes encoding the SP proteome display signs of a high rate of adaptative evolution [3,31,37]. Other components, including exosomes (epididymosomes, prostasomes and vesiculosomes) [38,39,40] and small-size proteins/peptides, such as cytokines [41] and enzymes with anti-oxidant capacity [42], are yet to be fully understood regarding sperm selection, function and fertility signaling to the female [43], as well as regarding the long-term effects on the health of the offspring, regardless of the species considered [44].

This review attempts, by including our own results on various animal classes and model species, to critically summarize the current knowledge of the roles played by the SP components on ruling fertility and prolificacy in animal models and humans. The SP’s importance is hereby particularly discussed in relation to sperm survival, function and signaling to the female immune system towards fertility modulation, in vivo findings with major relevance for the development of diagnostic/prognostic biomarkers and the refinement of reproductive strategies. The review is comprehensive yet intending to avoid reiteration of the large volume of available literature elsewhere; nonetheless, it is written as comparative as we could.

## 2. The Composition of the Seminal Plasma: Comparative Aspects

### 2.1. The Building of an Ejaculate Defines the Diversity of Its Composition

**Figure 1:**
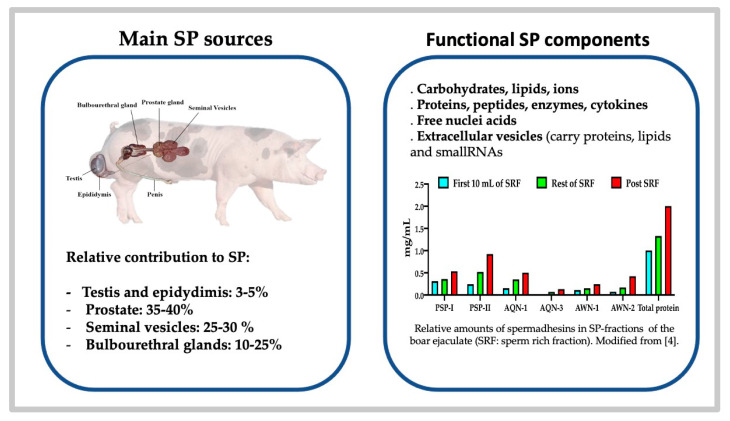
The sources, general functional components and variations in levels of the dominating protein, spermadhesins, along fractions of the ejaculated seminal plasma (SP) of pig, widely used by the authors as a biomedical research animal species.

Semen, e.g., the ejaculate, is a complex suspension of spermatozoa bathing in a heterogeneous composite fluid, the so-called SP, built by contributions of the testis, the epididymis and/or the accessory sex glands; the latter conspicuously lead—in direct relation to variations in presence and gland size among animal classes and species—to differences in the volume of the ejaculate and SP composition [24,45]. In some species (as in pig), the SP builds up most of the total ejaculate volume while the cell components (spermatozoa, other classically named round cells (lining cells of the excurrent ducts, epididymis or accessory glands, migrating leukocytes and spermatogenic cells), as well as extracellular vesicles (EVs), represent barely 5% (including a minor volume of epididymal cauda fluid) [4]. Ejaculation, a basic physiological phenomenon, differs among species in timing and ejaculate volume and type [24]. Of comparative interest, differences in ejaculate volume among species seem to relate to the type of mating and the site of semen deposition. Why? Basically because female anatomy rules, with ejaculation encompassing characteristics of the female genital anatomy that, in turn, defines the mating behavior [46] and site of deposition of the semen, i.e., cloacal or vaginal deposition for low-volume ejaculates with highly concentrated sperm suspensions. Some animal classes, such as poultry, lack accessory glands [47] and thus their ejaculate is very small, yet highly concentrated in a fluid built by secretions from the testis, the rudimentary ductus epididymis and the ductus deferens [47,48]. Ruminants, also having an un-fractionated type of sperm-dense ejaculate, and builds their SP by the concerted emission of cauda epididymal contents and of the secretions of a complete series of sexual glands (prostate, seminal vesicles and bulbourethral glands) [24]. They deliver an ejaculate produced during a very quick mating (seconds) by one-time emission of spermatozoa and sexual glands secretions. In primates and some species of ungulates, semen deposition is done deep in the vagina, in front of the cervical opening or in the vaginal fornix, while in others, sperm deposition occurs intra-cervically (pigs) or even intrauterine (equine) [24]. The latter species have voluminous ejaculates with less concentrated sperm suspensions. These differences are more intricate; these ejaculates are formed by cycles of sperm emission from the cauda epididymis combined with a sequential secretion and excretion by the sexual accessory glands [24]. Moreover, dogs, horses, pigs and men have fractionated ejaculates either because there is a dominant sexual gland (prostate in dogs) or an uneven blend of the emitted spermatozoa (still contained in the fluid of the cauda epididymis) with secretions of the accessory glands, verted in sequential spurts to the urethra [24]. In pigs, to cite the animal model most intensively studied by us, the SP is sequentially built by epididymal caudal fluid and the concerted secretion of the accessory sex glands: the prostate, seminal vesicles and bulbourethral glands [24,49] (Figure 1).

Common to boar, stallion and to some extent dog or man, the ejaculate is sequentially expelled in fractions usually classed as pre-sperm, sperm-rich (SRF) and post sperm-rich, clearly defined by the amounts of spermatozoa present and the volume and type of secretion building the SP of each fraction [4,11,50]. In general, the first secretion (pre-ejaculate) present in the urethra derives from the urethral and/or bulbourethral glands (Littré and Cowper for human), containing mainly mucin, sialic acid, galactose and salts in a slightly viscous, clearly aqueous fluid). The emission of spermatozoa from the cauda epididymis to the urethra, accompanied by secretion from the prostate, initiates ejaculation (e.g., expulsion of semen into the female or into a collection vial) in a series of clearly distinct spurts. In humans, the initial spurts are usually called the SRF of the ejaculate [51], since they hold the most spermatozoa, with a blend of the acidic cauda epididymis and ampullar fluids mixed with the slightly acidic citrate and zinc-rich prostate fluid, which also contains specific peptides and proteins, such as acid phosphatase and kallikrein 3—a prostatic specific antigen (PSA) in humans) [52]. In the following spurts, building the so-called post-SRF, there is a gradual dominance of secretion from the seminal vesicles (rich in fructose, peptides, proteins, prostaglandins (PGs), etc., which is clearly basic in nature) as well as gradual diminution of sperm numbers [52]. Presence of prostaglandins or steroid hormones, such as estrogens, in the post-SRF (vesicular-derived) has been well documented, with large species variation [24]. In pigs, fractions are also classically pre-SRF (with a clear sperm-free seminal fluid that contains mainly secretion of the urethral and bulbourethral glands, as well as the prostate), the SRF (composed by the emission of portions of the cauda epididymal contents, extended in vesicular but mainly prostate gland secretions) and, finally, the post-SRF (where the fewer emitted spermatozoa are largely extended in secretions of the vesicular glands, the prostate and, by the end of the prolonged ejaculation, of the gel-rich, coagulating bulbourethral gland secretion (Figure 1)) [11].

Equines follow a similar pattern of ejaculation [53] as pigs. In either species, during the gel-rich secretion, noticeable during collection with an artificial vagina, the gloved-hand technique can virtually coagulate the entire ejaculate if placed together. In vivo, the gel fraction in these species enters the cervical canal by the end of ejaculation, a process also seen in rodents to hinder other males from deposing their semen on the already mated female. In humans, at or immediately after ejaculation, a sample of semen collected in a single vial also coagulates to form a gelatinous mass that immobilizes the spermatozoa [24,54]. If an ejaculate is collected using a split procedure (i.e., several vessels for collection of different fractions), as it presumably occurs during ejaculation in vivo, the first spurts (prostate-dominated) do not coagulate, while the last ones (vesicular-dominated) do [54]. Such coagulum is rapidly (in vivo, within minutes) or more lengthy (15–30 min in vitro) liquefied by prostatic-derived proteolytic enzymes [55]. In livestock, the gel-fraction is routinely discarded during the ejaculate collection. Noticeably, an initial sperm-peak portion is present in the first 10 mL of the pig SRF, where a vanguard sperm sub-population of about 25% of the total sperm numbers [4] seems to contain, in vivo, the first and main colonizers of the sperm reservoir in the oviduct [26]. The phenomenon seems conserved across species [56], since most human spermatozoa are, as described, present in the first (non-coagulating) fractions, so a certain proportion of them can rapidly enter the cervical canal, as extrapolated from studies that recorded sperm present in the Fallopian tubes as early as a few minutes after coitus [57].

Differences in ejaculate building among species reflects on the SP composition. The SP is basically formed by secretions of epithelial cells and thus contains electrolytes, protein and steroid hormones, sugars and proteins/peptides, including enzymes [24]. The relative proportions of these components certainly varies among species, the year [43,58] and the type of ejaculate in question. Classical examples of the latter are when either all components are ejaculated at once (e.g., poultry and ruminants) or in relative different proportions (e.g., humans, stallion, boar, etc.). For instance, the porcine pre-SRF-SP is rich in Na and Cl; the SRF-SP contains proteins, steroid hormones, glycerophosphorylcholine, fructose, glucose, inositol, citrate, bicarbonate and zinc; while the post-SRF-SP has the highest amounts of proteins, bicarbonate, zinc, Na, Cl and sialic acid [4,24,49,59]. Although different ions of the SP seem to play important roles in maintaining sperm survival and function [43], with bicarbonate modulating sperm motility or destabilizing the plasmalemma [60], or zinc as modulator of chromatin stability [61], most other roles, including those related to fertility, are connected to its large protein contents (human 25–55 g/L; poultry 7.5–9.0 g/L; boar 30–60 g/L).

### 2.2. The SP Proteome, What Does It Contain and What Does It Impact?

The SP of most species contains protein compounds similar to those present in blood plasma, such as pre-albumin; albumin; α-, β- and γ-globulins; transferrin; enzymes and some immunoglobulins; complement factor; as well as differential amounts of cytokines and chemokines [11]. Comprehensive sperm protein databases have been established for a plethora of species, including insects, avian, ruminants, porcine, equine, or human [11,32,34,62,63,64,65]. The sperm proteome covers the expected spectrum of function (from energy production to cell recognition), but few proteins are accurately linked to (in)fertility [66]. The seminal fluid proteome, even in species lacking all accessory sexual glands as in avian species [64,65], depicts clear phylogenetic relevance for fertility for its direct relation to sperm function, mechanisms of energy production/consumption, proteolysis and oxidoreduction.

In mammals, SP proteome studies have dramatically expanded over the past decade, allowing for the identification of large and small proteins and peptides, which now account for the thousands available [35,67,68,69,70]. The main SP proteins belong to one of three groups: proteins carrying fibronectin type II (Fn-2) modules, spermadhesins or cysteine-rich secretory proteins (CRISPs) [71]. However, differences in type and source of proteins are present among species, owing to the already named differences in glands and/or the sequence they are emptied, or the type of ejaculate they have. Comparative studies [63] have shown particular roles played by SP proteins in ruminants amply used for AI, holding single ejaculates [66,69,72,73], but also in species—including humans—with fractionated ejaculates, where the proteome type, concentration and temporal exposition are relevant [11].

The human SP has thousands of unique proteins, ~25% secretory from the accessory glands [74], and either free or present in epididymosomes and prostasomes [75,76,77]. The prostate secretion, in humans representing 20–30% of the total SP volume, and even more in other species where the gland is solely present (e.g., dogs), is the first SP portion to confront the cervical canal, being in immediate direct contact with the major numbers of emitted spermatozoa. In humans, three major proteins, all under hormone regulation, have been identified: the kallikrein PSA (mainly released by the prostate but also produced by the Littré glands), the prostatic acid phosphatase and the cysteine-rich prostate-specific protein-94 (PSP-94, β-inhibin-β-microseminoprotein) [68,74]. The primary function of PSA is the liquefaction of the coagulum by hydrolyzing the majoritarian semenogelins I and II, involved in the gelification of the latter spurts of the ejaculate (coagulum), while prostatic acid phosphatase and the PSP-94 have enzymatic growth factor action. The liquified coagulum contains products with clear biological functions, such as inhibition of sperm motility, antibacterial activity, etc., alongside with other seminal vesicle proteins that include lactoferrin, fibronectin and protein C-inhibitor [68,74]. The Cowper’s gland (which is difficult to sample as isolate) contains an extremely abundant protein: mucin [24].

The stallion displays not only a similar fractionated ejaculate as humans [78], but shows equivalent main SP proteins, such as HSPs (horse seminal proteins 1–8), Fn-2 and CRISPs [79]. Most HSPs are of low molecular weight (14–30 kDa), form multi-protein aggregates, and all but HSP-4- are capable of attaching to the sperm surface. The prevalent HSP 1–2 short Fn-2 type heparin-binding proteins (70–80% of the total protein) modulates capacitation, changing the sperm membrane structure [80]. Studies of isolated ejaculate fractions showed HSP-1 was the major protein present in all, while the first fractions contained acrosine inhibitor, prostate-specific antigen (PSA) and PSA and other kallikrein-family proteins (HSP-6 and HSP-8), and the other HSPs were present in the rest of the fractions [53]. The equine CRISP-3 (HSP-3) is associated with fertility [81], probably in relation to the tolerance of spermatozoa to preservation [82], as it was shown it occurs in a dose-dependent manner [83].

**Figure 2:**
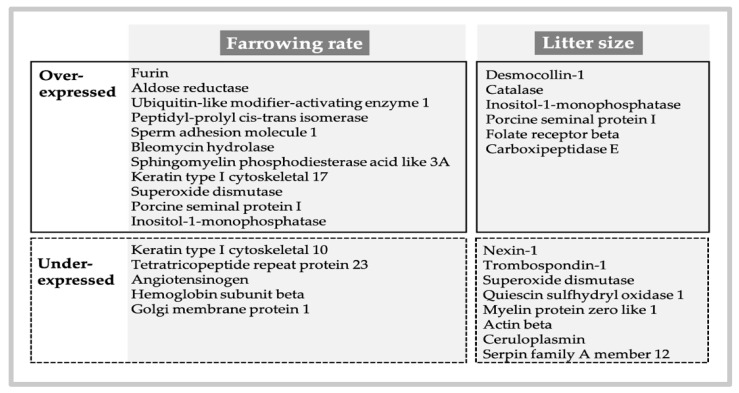
Over- and underexpression of less abundant specific seminal plasma proteins in the animal model pig (*Sus scrofa*) analyzed using iTRAQ-based quantitative proteomics and their relation to fertility, as farrowing and prolificacy (litter size) (modified from [33,96,97].

Another species with a fractionated ejaculate, whose delivery sequence resembles humans, is the pig, our most commonly used animal biomedical model [84]. Of interest, likewise to humans, the porcine SRF has a sperm-peak portion where 25% of all the ejaculated spermatozoa are present within a volume of 10 mL, i.e., 1/20 of the total ejaculate [4]. This sperm-peak portion appears as the vanguard sperm sub-population, which firstly negotiates female barriers and colonize the oviductal sperm reservoir [26]. This sperm-peak portion does not coagulate (alike human) and it is rather protein-poor, yet containing epididymal lipocalins and an inhibitor of acrosin/trypsin [4]. In contrast, the latter ejaculated fractions contain increasing amounts of SP proteins, verted by the vesicular glands (Figure 1). Between 75 and 90% of these SP proteins belong to the multi-functional spermadhesin lectin family of 12–16 kDa glycoproteins (the heparin-binding HBPs Alanine–Glutamine–Asparagine proteins AQN-1&3, the Alanine–Tryptophan–Asparagine proteins (AWNs) and the non-heparin binding porcine seminal plasma proteins I and II (PSP-I and PSP-II) [4,11,22,32,85]. These spermadhesins, the PSPs in particular, attach sequentially to the sperm plasma membrane from the testis to the ejaculate [86], promoting sperm survival through membrane stabilization [87], and fertilization capability [87,88] by modulating capacitation and sperm-oviduct/oocyte interactions [85,86,89,90,91]. Moreover, they appear to regulate the timing of ovulation [92] and further showing immunostimulatory activities in vitro and in vivo, presumably in relation to specific cytokines [4,11,25,93]. Besides spermadhesins, a plethora (several hundred specific to *Sus scrofa* taxonomy) of other less abundant pig-specific SP proteins are present [32,34], yet only a few influence the reproductive processes. Noteworthy, their presence and degree of expression relate to specific ejaculate fractions, associating quantitative variation to the effects depicted by the SP of these fractions, particularly the SRF vs. the post-SRF [2,32,94,95]. Up to 16 SP proteins represented in the *Sus scrofa* taxonomy were differentially expressed between the SRF and post-SRF. Eight of these, overexpressed in SRF, had been previously related to sperm membrane function, capacitation, the acrosome reaction and zona pellucida binding [32,33]. A follow-up study, using a pre-fractionation step by solid phase extraction, detected further numbers of less abundant SP proteins in boars with different farrowing rates and litter sizes after AI of 10,526 sows. Here, there was an overexpression of SP UBA1, SPAM-1, AKR1B1 and furin in high-fertile boars and of CAT and DSC-1 among boars registering large litter sizes, clearly confirming the relevance of the SP proteome for boar fertility [34] (Figure 2).

It is thus evident that the proteins of the SP exert multiple functions, starting with their general influence as a vehicle aiding sperm survival when semen is highly extended [43,87] or processed [22,98,99], to specific events while adsorbed to the sperm plasma membrane, reinforcing its stability during uterine sperm transport [100]. Besides, specific SP-proteins have pro-inflammatory capacities, contributing as a trigger for the well-known post-mating primary, transient inflammation whose role is to cleanse the intra-uterine lumen from foreign cells, proteins and eventual pathogens, as well as from excessive spermatozoa, e.g., those not involved neither in colonization of the tubal reservoirs or fertilization. Moreover, SP-signaling is, as we shall later see, also involved in long-lasting signaling to the female, towards a more immunotolerant environment in preparation for the descending embryo and the triggering of a long-lasting immune modulation towards tolerance of the hemi-allogeneic embryos/placenta bearing paternal antigens [11,101,102].

Interestingly, the roles of the seminal fluid proteins mentioned for these mammals seem to be highly conserved across animal classes. The transfer of seminal proteins induces particular post-mating changes in female insects, inducing changes in behavior regarding feeding and decreased receptivity for re-mating, as well as of gene expression of the mating-dependent genes [103]. Such changes in gene expression can modify metabolism and enhance egg production, modulate sperm storage and competition, or trigger expression of antimicrobial peptides for immune defense [104]. Our own studies in phylogenetically distant species, such as chicken and pigs, has confirmed that exposure to SP proteins in vivo induces gene expression changes, affecting both the innate and adaptative immunological responses of the female, in direct relation to sperm survival and fertility [31,105].

### 2.3. Cytokines in the Seminal Plasma: The Effectors of Such Signaling?

The SP of most species contains differential amounts of a 5–20 kDa peptidomes with pro- and anti(or tolerance-related)-cytokines and chemokines [11]. Both in human and boar, cytokine amounts of both inflammatory (tumor necrosis factor (TNF)-α, interferon-γ (IFN-γ), interleukins (IL) −6, −8, granulocyte-macrophage colony-stimulating factor (GMCSF)), and anti-inflammatory cytokines (thymus- and activation-regulated chemokine (TARC), macrophage colony-stimulating factor (M-CSF) as well as transforming growth factor-β (TGF-β)_1–3_) were higher in the vesicular-dominated fractions [11,41,106]. This relation between cytokine concentrations and SP-fraction was consistent among the TGF-β_1–2_, IFN-γ and IL-6, IL-10 and GM-CSF [41,106,107,108,109]. In the human and pig, cytokines relate to sperm viability, semen function and hence fertility [41,70,95,110]. As well, cytokines—probably in concert with other SP proteins [11,111]—play a role in modulating the uterine immune-cytokine network [6,88,112,113,114,115,116,117], to facilitate the transitions in immune responses by the female mentioned earlier and what appears central for successful fertility [93,102,118,119,120]. In this context, it is also worth noting the function of the adipokines secreted by white adipose tissue. Adipokines have, from being considered endocrine regulators of energy metabolism and secreted by white adipose tissue, evolved as involved in fertility regulation, perhaps acting as metabolic sensors [121]. Leptin and adiponectin are now accompanied by the novel adipokines resistin, chemerin, apelin and visfatin, which, alongside cognate receptors, are now evidently expressed in the reproductive tissues of human and animals, including the testis [121], and affect various reproductive events [122]; their disarray or absence can also cause infertility [123]. Being produced by the testis or incoming into the male genital tract, they have been identified in SP in humans and animals, including the pig [124,125], and they attach via specific receptors to the sperm plasmalemma [126]. Their impact appears related to sperm morphology, function as well as being involved in inhibiting capacitation, or in inflammatory processes in infertile individuals [127]. Noteworthy, levels of some adipokines in SP, such as leptin, are 10-fold higher in the boar than in humans [125].

### 2.4. Enzymes of the Seminal Plasma: Do They Play a Role in Fertility or Are They Simple Markers of Sperm Function?

In general, about 3% of proteins in SP are enzymes [70], with some related to sperm quality, such as lipases, matrix metalloproteinases (MMPs) and glycosidases (β-glucuronidase (BG), α-glucosidase, β-glucosidase, α-galactosidase, β-galactosidase and β-N-acetylglucosaminidase (NAG), etc.) [24]. The pig SP contains also several antioxidative enzymes, such as superoxide dismutase (SOD), catalase (CAT), gamma-glutamyl transferase (GGT), glutathione peroxidase (GPx)/phospholipid hydroperoxide glutathione peroxidase, glutathione reductase/S-transferase and paraoxonase type 1 (PON-1), displaying the highest levels in the fractions of the ejaculate containing most spermatozoa [43]. Most of these enzymes alongside antioxidants such as glutathione, ascorbate, pyruvate, taurin and vitamin E (α-tocopherol) in SP protect spermatozoa against excessive levels of reactive oxygen species (ROS), which could lead to lipid membrane peroxidation and consequently in sperm damage and death [128]. This protective action against ROS damage is reflected in the fertility of the semen, particularly after processing [129]. Particular enzymes appear significantly associated with the fertility data of males, including the epididymal lipocalin-type PGD_2_ synthase in equines [130,131] and pigs [94], the PON-1 [132]; or the lactate dehydrogenase isoenzyme LDH-C4 [133] also in the pig.

## 3. The Particulate Seminal Plasma: The Most Relevant Component of Seminal Plasma?

**Figure 3:**
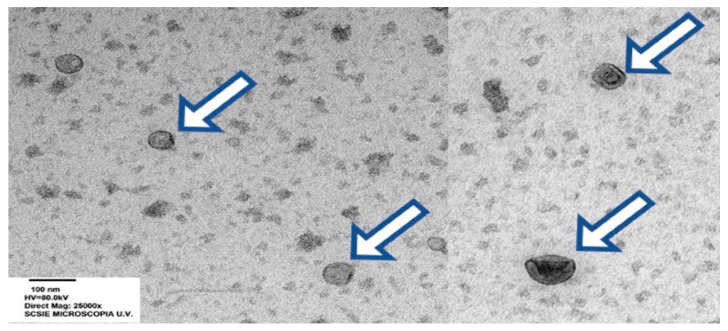
Transmission electron micrographs of extracellular vesicles (EVs, arrows) in porcine seminal plasma (bar: 100 nm), courtesy of Barranco et al. [39].

Having reached this section in this review essay, it appears confirmed that the SP is evidently far from being a simple fluid, as it contains diverse molecules. Analysis of a tube containing a collected ejaculate that has been spun at relatively low speed/time to form a pellet of the suspended spermatozoa and eventual somatic cells revealed—using electron microscopy, for instance [134]—a fluid with a large number of membrane vesicles of various dimensions, aspect and, particularly, origin (Figure 3).

**Figure 4:**
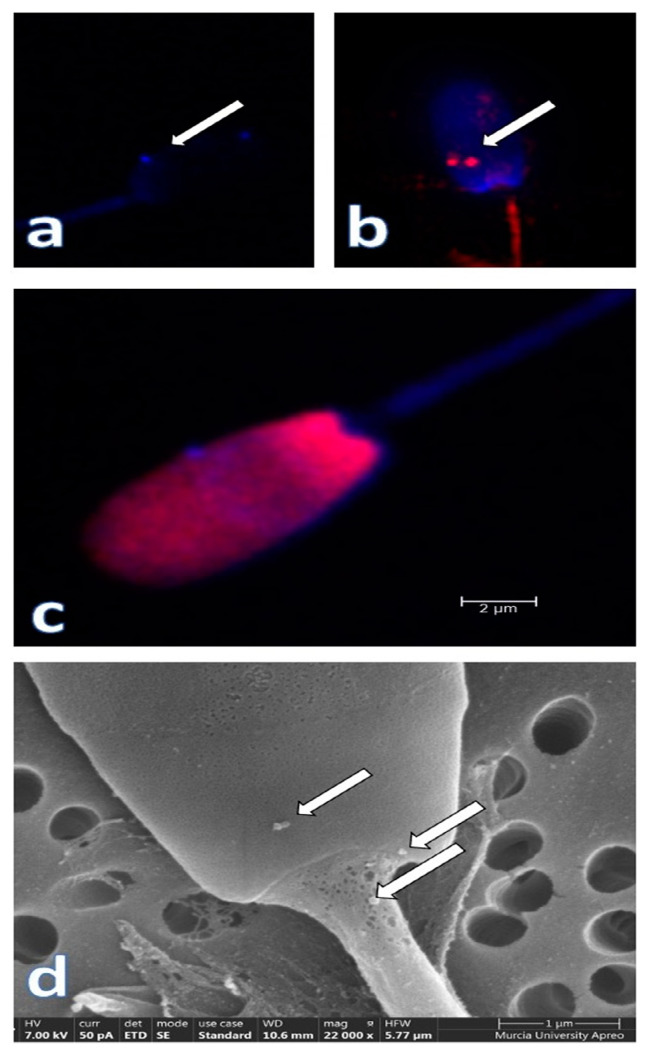
Seminal plasma extracellular vesicles (EVs) attached to the plasmalemma of ejaculated pig spermatozoa (arrows). Confocal microscopy microphotographs show the CD63 (**a**) (blue, Alexia fluor 405) and CD9 (**b**) (red, phycoerythrin, arrows) immunostained EVs. In (**c**), a composite image of a spermatozoon stained with Hoechst 33,342 and propidium iodide, while a scanning electron microscopy (SEM) micrograph showing some EVs attached to the membrane in the neck and head sperm domains is shown in (**d**). Courtesy of our graduates L Padilla and I Barranco.

These EVs are shed by the different organs that contribute their secretion to the seminal plasma, namely, the testes, ducti epididymis and the accessory sexual glands, explaining why they display diverse cell-derived membrane structures. These EVs sequentially interact with the spermatozoa (Figure 4) in their journey towards ejaculation and, when ultimately within the female genital tract, interact with its epithelial lining.

Such interactions with the female are well reviewed in reference [135], and the large number of references therein. EVs are conserved structures along humans [136] and animals [39,40,75,137,138,139,140], and thus they are prompt to explain how influential they might be for crucial events in reproduction. Just consider the fact that the most relevant of these lipid bilayer nanovesicles, either being exosomes (processed multivesicular bodies released by exocytosis, of 30–100 nm diameter) or the 100–1000 nm size out-budded plasma membrane microvesicles), encapsulate a rather complex load of lipids, signaling proteins, small non-coding and regulatory RNAs [134,140], which basically define the SP EVs as information carriers from the producing organs to specific targets, these being spermatozoa or the female internal genital tract. Their role is to modify the functions related to sperm motility [141], capacitation [142,143,144] and perhaps fertilizing capacity, or even the immune responsiveness of the female against paternally derived antigens [102]. These active biomolecules are better protected from degradation and loss-of-function within EVs than the free SP-molecules are, an aspect that we should seriously consider if we aim to find relevant biomarkers for (dys)function [145] or use as signaling components, or even as particulate therapeutic additives [146].

However, let us go back to the fact that the SP is built by several secretions and, moreover, the fact that spermatozoa are exposed to EVs during one of the most crucial periods once shed from the seminiferous epithelium: sperm maturation in the epididymis [147]. During this journey, lasting days, the spermatocrit is concentrated, and the spermatozoa sequentially gain capacity to move forward and to fertilize [148,149], to be finally stored, quiescent, in the epididymal cauda [147,150]. During this processing, the spermatozoa are exposed to electrolyte changes, to an hyperosmotic and increasingly acidic milieu [150], but, noteworthy, also to the bombardment of 20–250 size EVs (named epididymosomes, [75,151] that are shed by the lining principal cells and that modify their cargo of protein [152,153], which includes antioxidative enzymes [42,129,132,154,155] as well as their phospholipid and cholesterol content [156], conveying an extensive lipid remodeling [157,158] that would stabilize the plasmalemma but also prepare for the destabilization changes that occur during capacitation. The EVs also contain a very particular load of ncRNA (particularly miRNAs, [140,159,160], a matter we shall discuss later. Epididymosomes are tethered to receptors on the sperm plasma membrane via proteins as tetraspanins [161] or cell-surface glycoproteins involved in cell-to-cell interaction, such as CD44 [40]. These proteins are up to now mostly used as markers [39,40] despite their roles in the biogenesis, cargo selection and cell targeting of the EVs, or in cell contact and uptake [161]. Considering the latter, CD44, suggested as an eventual marker for epididymosomes [40], is the main receptor for hyaluronan, the most conspicuous glycosaminoglycan present in the oviductal sperm reservoir and the expanded cumulus cells [105], and recognized as interplay between the spermatozoa and its surroundings in crucial events such as colonization of the tubal sperm reservoir or capacitation, as well as sperm location of the oocyte [105]. In sum, it appears evident that epididymosomes convey fundamental mediation during sperm maturation [162] (Cornwall 2009), promoting acquisition of sperm motility, oxidation–reduction, metabolism, capacitation, acrosome reaction and fertilization [137,152,153,163,164,165]. Alongside, there seems that the epididymosome load of ubiquitin [166] or ELSPBP1 (epididymal sperm binding protein 1) [167] aid tagging defective or dead spermatozoa that are to be eliminated along the journey or after ejaculation [152,166,167,168]. Epididymosomes are also contained in the caudal fluid that accompanies the spermatozoa emitted during ejaculation [40,169]. In species with fractionated ejaculates, the first portion/s is/are often called the sperm-peak portion [4], being the one primarily extended with prostatic fluid and the first to enter the female genital tract and whose fortuitously contained spermatozoa are even overrepresented in the oviductal sperm reservoir [26]. Obviously, epididymosomes can modify both ejaculated spermatozoa [40,75] and/or the influence the female genital tract [27,40,75,102]. In chickens, where a rudimentary epididymis is prevalent, we have not been successful in detecting relevant amounts of epididymosomes in seminal fluid, at least not using the same tetraspanin/CD44 markers we used in pigs [170]. In turn, a very recent study, using ultracentrifugation, the WB of the protein markers and EM has shown the presence of small EVs (possibly exosomes), in the seminal fluid of roosters with different fertility with capacity to putatively incorporate into spermatozoa [171]. Our own studies using pig as model animal have shown that specific fractions of the ejaculate or even their sperm-free SP elicit dramatic modifications to the expression of genes related to sperm survival and function as well as to the immune status of the female’s various compartments of the internal genital tract—pre-ovulation [29,172] but even during early embryo development [173]. We have seen a similar set of results in chicken in response to mating, despite the uncertainty of the presence of similar types/amounts of EVs in roosters [170], implying the response might well be a conserved mechanism in animals with internal fertilization [3]. Since there is a mix of EVs shed by the prostate or the seminal vesicles (or even perhaps the bulbourethral glands) [76,169,174,175], pig SP EVs show heterogeneity in surface markers [39,40,140], probably because they also have a unique high level of cholesterol and sphingomyelin [143] and of lipoproteins (LDL/HDL) [40], which, by stabilizing the plasma membrane, could later aid in regulating sperm membrane fluidity before and during sperm capacitation [56]. As mentioned above, the SP EVs have demonstrated capacity to bind to and even fuse with the sperm plasma membrane to deliver their cargo [76,138,176,177], influencing sperm function [142,143,144], but also via proteins such as CD59, to protect spermatozoa from responses by the immune system [135]. SP EVs have also proven, as did other EVs [178], to influence the local immunity of the female internal tract [179], and to induce inflammatory and immune responses by pig uterine cells [4,38,180]. Whether the changes in immune-related genes we have detected in peri-ovulatory sows following mating or AI, with selected ejaculate fractions (particularly the sperm-peak portion) or their sperm-free SP [29], or even in sows having pre-implantation embryos [173], are conveyed mainly by free molecules, by SP-EVs or it is the effect of a concerted signaling, remains to be experimentally tested. Such exploration of the action of the epididymosomes, prostasomes and vesiculosomes is possible using a proper experimental design, in pigs and in other species with fractionated ejaculation, since the various fractions containing major subpopulations of EVs can be manually collected. Their separate harvesting and characterization, if possible, could make possible the design of experiments for their separate roles on spermatozoa and in females, in vitro or in vivo. The prospect of using EVs as biomarkers for fertility or additives for improvement is exciting, considering that the use of crude SP or even isolated proteins/enzymes suffers from a short functional lifespan often caused by nearby proteases.

There is increasing evidence that the load of non-coding, smallRNA molecules in semen differentially contributes to reproduction, and that the dysregulation of their expression can explain many dysfunctions associated with infertility [181]. Small RNAs are present within spermatozoa [27] as well as in the SP-EVs [140] and most likely free in the SP, or bound to membrane structures (of the spermatozoa, other cells or cell debris). These RNA molecules are part of the set of paternal RNAs whose transcriptional origin differs in between. Some of these were originally transcribed in the testes and retained selectively in spermatozoa entering the epididymis; another set were RNAs of epididymal or accessory gland origin that were added to the spermatozoa via EVs. Finally, we have paternal RNAs that, enclosed in EVs or free in the SP, further directly interact with the female internal genital tract.

Processing of the collected semen is of utmost importance since it can surely mix up the different allocations of the small RNAs being detected. Many small RNAs have been explored and detected in the male genital tract and semen, including microRNAs (miRNAs), PIWI-interacting RNAs (piRNAs), YRNA and transfer RNA (tRNA)-derived small RNAs (tsRNAs) [136,182,183,184]. The most commonly explored small RNAs are the single-stranded 22–24 nucleotides miRNAs, considered important regulators of post-transcriptional gene expression [182,185] and detected in the testicles, spermatozoa, free in the SP and within SP EVs [181]. The secondly most found are the piRNAs, built by 24–31 nucleotides, which are able to bind to the PIWI proteins to modulate spermatogenesis [186]. Moreover, there is evidence that circular RNAs (circRNAs) are also present in spermatozoa [187], including the pig [188]. The small RNAs present in boar semen are particularly detected within spermatozoa, accounting for about 1% of the sperm total RNAs, and include miRNAs, tRNA fragments, piwiRNAs, miRNAs and YRNAs [182], but also the above mentioned circRNA. The latter appears to be an in vivo long-lived (24 h) RNA molecule owing to its closed-loop structure, which can both act as a sponge for miRNAs, protecting them from degradation, but even be related to sperm phenotypic variables, such as sperm motility [188].

Differential analyses of the pig sperm transcriptome have shown the relevant associations with sperm quality [189] and fertility [27,190], confirming data in other species [182,191,192,193,194]. The SP EVs also contain diverse smallRNAs, among which the majority are messenger RNA fragments (mRNA, about 25% of the total reads) followed by piRNAs (15%, almost 20,000), miRNAs (9%, almost 300) and very small amounts of tsRNA (0.01%) [140]. The most abundant, likely functional miRNA was ssc-miR-21-5p, which is related to the inhibition of the VCL gene that, by constraining sperm capacitation, is considered to negatively affect fertility [195]. The other most common miRNAs were ssc-miR-148a-3p, ssc-miR-10a-5p as well as ssc-miR-200b and ssc-miR-10b [140], the latter present within spermatozoa and in SP EVs [189]. Such double location reinforces the need of considering the role of EVs as adding material to the spermatozoa yet reminding us of the relevance of proper isolation of the components of semen, so that they can sequenced separately but compared at the same time. Unfortunately, sample sizes are uneven, and semen from boars with well-studied and established fertility must be primarily studied [190], a shortage nowadays. However, how are these miRNAs affecting fertility? Directly, by affecting sperm quality, of course [196]. However, for some species, such as bovine or particularly porcine, breeding males are selected for fertility and discarded when their AI-results are compromised, even when their sperm quality surpasses the established thresholds for normality [197]. So, what could these miRNAs rule that impacts fertility? As already mentioned, SP has the capability of eliciting inflammatory and long-lasting immune signaling by the female genital tract [27,29,117,173], being even able to cause release of miRNAs from the lining epithelia [180]. Responses from the female can be issued by spermatozoa alone [198] or even by the act of copulation [199], but the evidence is that these changes are rather modest, compared to what the SP can elicit, either alone or as semen (i.e., including the suspended spermatozoa). Many of the top miRNAs detected in pig spermatozoa [27] and SP EVs [140] can impact TLR4 signaling [200], and promote the production of cytokines that modulates immune responses [27,180,201] to the extent of modulating the fertility of the males considered [27,190].

## 4. What Proof Do We Really Have That Seminal Plasma Really Affects Fertility?

**Figure 5:**
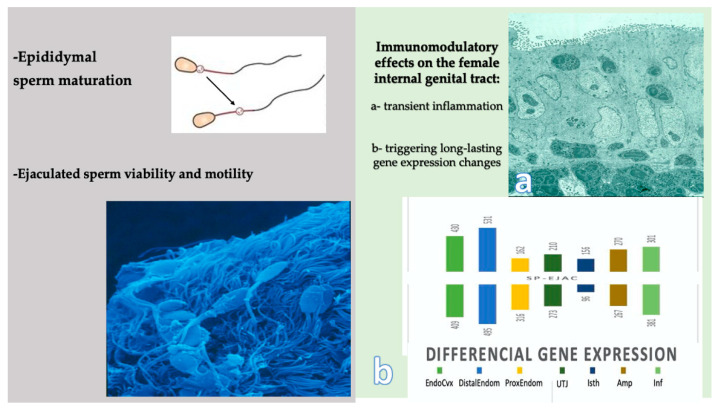
Major recognized functional effects of seminal plasma (SP) in vivo. In the male (left panel), the cytoplasmic sperm cytoplasmic droplet is lost at ejaculation, and SP promotes forward motility of the viable ejaculated spermatozoa along the female genital tract. In the female (right panel), SP elicits immunomodulatory effects in the genital tract, firstly through a migration of leukocytes from the lamina propria through the epithelium during an initial transient inflammation (**a**), concomitant with a triggering of long-lasting differential gene expression changes in the female (**b**): EndoCvx: endocervix; Distal-Prox Endom: distal/proximal endometrium; UTJ: utero-tubal junction; Isth: isthmus; Amp: ampullas; Inf: infundibulum.

We have assumed that the fertility of a sire is not only ruled by the spermatozoa but also by the SP. However, can we separate these two intrinsic components of semen? One could state that this is what has been proven when IVF was established, putting most weight on spermatozoa, which included the removal of the native SP. IVF and intracytoplasmic sperm injection (ICSI) of ejaculated or epididymal spermatozoa, or even elongated spermatids that have never gone through sperm maturation, has resulted in fertilization, embryo development, pregnancies and births in humans [202] and animals [203], implying that the SP is not a mandatory component. However, human fertility (as baby births) after embryo transfer of IVF/ICSI “fertilized” oocytes has been steady around 30% since 1994, in general terms [204]. Such a steady low efficiency has, together with experimental evidence of the effects of seminal plasma on the female, called for the application of SP during or after human embryo transfer [205,206,207]. Growing evidence has established SP playing major roles in embryo development and birth rates [102], and although the matter remains unsolved for humans, the experimental evidence from various animal models has quite nicely defined these effects on the female [12,93,120] (Figure 5).

The composition of SP and the differences among the ejaculate fractions has help define the possible influence on the fertility of the male [88,96] via interactions with the female genital tract [27,29,208,209]. Fractions seem to vary in their capacity to elicit inflammation, i.e., the porcine post-SRF fraction, by containing higher amounts of SP-proteins, particularly of the PSP-I/PSP-II, is capable to induce entry of polymorphonuclear leukocytes to the uterine lumen within minutes of exposure [25] (Figure 5). Thus, the post-SRF is considered as less permissive for fertility, compared to the SRF that is “less” protein-rich, and contains many of the proteins and peptides present in the sperm-friendly caudal epididymal fluid [4,11,210]. However, one should not forget that the situation in vivo—particularly for species with fractionated ejaculation and semen deposition intra-cervix or utero—is far from the analysis of an ejaculate in a test tube.

Do we have evidence that the SP per se is, by inducing the changes of gene expression in the internal genitalia of the female we have already pointed out, able to mark differences in fertility when added separately from spermatozoa? The answer seems to be yes, as rates in early embryo survival [88,93,102,120,173,208], implantation [101,102,115], placental development [116], farrowing, litter size and even offspring development and health [44] could be improved by additional inseminations with homologous/pools of SP.

**Figure 6:**
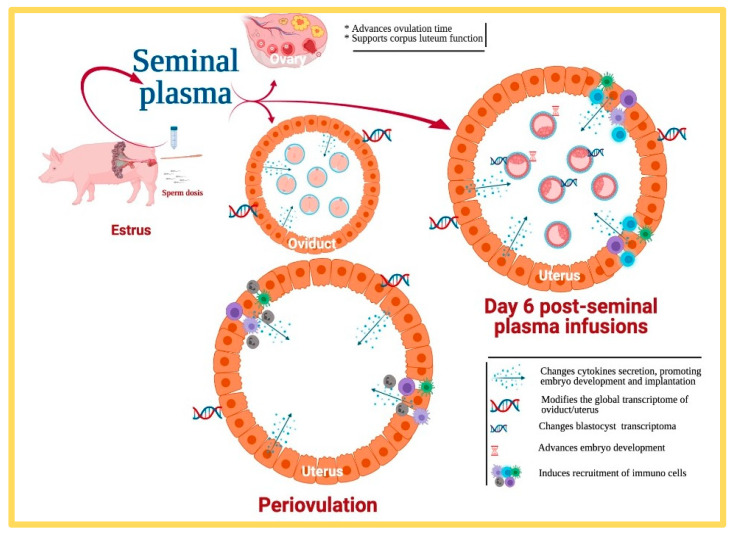
Infusions of additional sperm-free pig seminal plasma during estrus causes a plethora of functional effects in the female, from advancing ovulation, supporting corpora lutea development, to a significant number of events six days later, at the oviduct and uterus.

Many of the above studies, and of many others being rather empirical in nature, however, show simple associations between the presence of SP and effects, with some of these complex in nature. The evidence gathered via association studies, at least regarding livestock fertility, show for proteins [11,33,35,36,43] or cytokines [41,70] either positive, negative or ambiguous statistical relations. This variation is not surprising, considering inter- or intra-male variations in composition and the relative amounts of these molecules and even the effects of handling [43,58,109]. Such variations, together with specific differences in experimental layout, are present among the plethora of contradictory reports [36,94,131,211,212]. However, this should not be a definitive hurdle, and finding a direct relation between a particular protein and fertility, despite remaining elusive, should be the purpose of future research to either secure suitable biomarkers for diagnostic/prognosis purposes or even the isolation of specific molecules to be used as additives. Associations are not definitory and, particularly, do not reveal mechanistic elements, calling for deeper experimental studies. We have attempted to recently disclose the role that native SP infusions during estrus (i.e., in relation to AI) could have on the modification of certain cytokines produced by the endometrium and released to the lumen (rather than focusing on the SP-cytokines) during the pre-implantation period. Such an estrus SP infusion has been tested earlier [213], revealing that several cytokines, such as GMCSF (relevant to early embryo development and present in pig genitalia) [108], were related to evident changes in pre-implantational embryo development, a matter we have confirmed; infusions of SP modifies the transcriptional pattern of the endometrium and advances embryo development in pigs [208]. Noteworthy, the uterine environment during the pre-implantational period in pigs seems to have a dominant anti-inflammatory cytokine profile [93] throughout, and the SP infusions definitely induce overexpression of genes associated with embryo development and implantation in Day 6 porcine blastocysts [173] (Figure 6).

Among the relevant genes overexpressed, SP-treated sows showed an overactivation of the transforming growth factor-ß (TGF-ß) signaling pathway, known as the pathways supporting the proliferation of Treg cells by regulating dendritic cell function [214] and thus increasing the development of Treg cells and helping control the immune response of the female in response to the presence of hemi-allogeneic embryos as early as Day 6 of pregnancy [215]. Since TGF-ß is recognized as present in boar SP [106,107,213] and its action has been tested in relation to litter size [216], the question arose as to whether its infusion at estrus would be responsible for the above changes seen. The experiment we performed compared intrauterine infusion of TGF-ß_1_ with native SP prior to insemination for their effects on both endometrial cytokines and on the development of pre-implantation embryos. It was rather evident that both SP and TGF-ß_1_ were able to influence endometrial cytokines, but only SP impacted pre-implantation pig embryo development, thus implying SP has a concerted action of various factors influencing embryo development by way of a modified gene expression by the female endometrium [120]. Finding out which particular factors are mechanistically most relevant seems appropriate and experimentally possible, albeit complicated. However, the prospect of finding a group of relevant biomarkers, and eventually additives, raises the bid.

## 5. What Else Can Be Done with Our Current Knowledge of SP?

### 5.1. Can We Improve Andrology Diagnosis by Using Specific SP Biomarkers?

Most likely; this is indicated by the many SP proteins that have been associated with decreased motility and viability, and further to fertility [32,34,63,94,217], since these would be very valuable for diagnosing which sires can be prevented or removed from breeding. With some components of SP being associated with (in)fertility, identification of the males bearing presence or most-likely higher amounts of some can aid their removal as breeding sires, paving the way for the use of more fertile ones, and thus via diagnostics improve fertility in the long run. Molecules showing association with (in)fertility are innumerable, but association between variables is not definitory of function in a general population. Markers for sperm dysfunction [218] and (in)fertility have been detected in spermatozoa [27,190,219,220] and in the SP [221,222] of many species, including humans [218,222,223,224,225] and livestock [70,226]. They vary from gene presence [227], markers of Sertoli cell function [228], proteins [36,229,230,231], anti-oxidative enzymes [223,232,233] and metabolites [234,235,236] to exosome-associated molecules [237,238] and microRNAs [181,239,240,241].

On the down side, the methodologies to identify these markers are rather complicated and still only available at specialized laboratories [97], but we have seen major developments to mark the presence or even levels of specific proteins using sensors, so that these methods will probably evolve for wider use. In addition, seminal plasma handling protocols would have to be standardized to achieve comparable results between laboratories [109].

### 5.2. Can We Enhance Sperm Function and Cryosurvival Using Seminal Plasma?

A parallel approach, complementing diagnostics for (in)fertility, is to identify molecules that enhance fertility in the semen of individuals already fertile. Yet, we need to remember that SP has been historically described as friend and foe to sperm survival. Being evolutionarily present, its roles for sperm function depend on what we consider. Spermatozoa are highly specialized cells, programmed for destabilization during their journey to the site of fertilization. They are covered by SP proteins whose adsorption to the plasma membrane prevent its destabilization and disruption, once gathered under the title of “decapacitation factors” [210], relevant for in vitro processing and in vivo survival [100,242]. However, this friendly role can soon change. For instance, if maintained in its native SP on a lab bench or even in a very simple medium, ejaculated spermatozoa will sequentially perish, mostly due to temperature changes, a lack of energy substrates and due to the action of the proteolytic enzymes in the SP and the dead cells, which will moreover change the osmolarity and ionic composition in the suspension. However, before this happens, many spermatozoa will interestingly capacitate [243], simply because many SP proteins shall aggregate and separate from the sperm plasma membrane and because phospholipid-binding SP proteins will induce continuous cholesterol efflux [244,245,246]. Consequently, for some species (pig and horse [247]), the SP is customarily removed during semen processing, but maintained for others, i.e., ruminants, where the problem seems minor, nonexistent, or its presence is considered vital [248,249]. The species differences accompany particularities in ejaculation and semen deposition in vivo; the first species having fractionated, intra-utero deposition, while the latter are vaginal depositors of a bulk, highly concentrated ejaculate.

**Figure 7:**
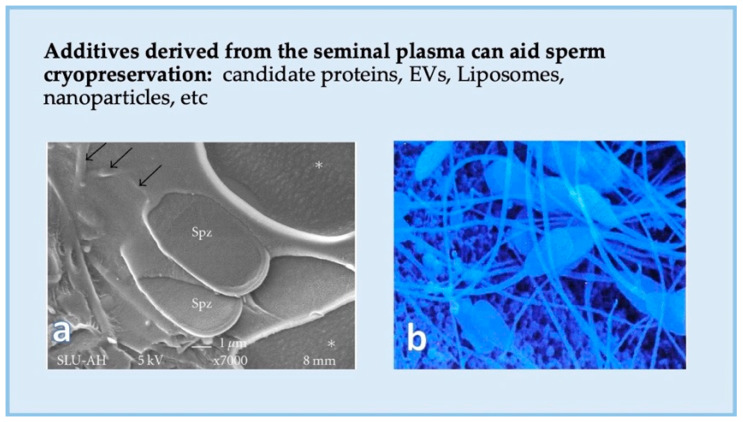
Additives derived from the seminal plasma can aid the cryopreservation of pig spermatozoa. Scanning electron microscopy (SEM) micrographs of (**a**) frozen (arrows show the frozen extender, embedding spermatozoa (spz and small arrows) as well as areas of frozen free water (*), Cryo-SEM), and (**b**) frozen–thawed boar spermatozoa.

Semen being processed, for any species, seems to benefit from the presence of SP for sperm survival and function, for instance when high-extension rates are applied [248,250,251]. In species with fractionated ejaculation of less concentrated semen, such as pigs and horses, SP is relevant when the semen is cooled [250,251,252,253,254], and particularly, when cryopreserved (Figure 7) [2,22,43,95,255], to the extent of affecting embryo development in vitro [256]. The impact of SP on freezability enabled not only the selection of males as good or bad freezers [70,257,258] but proved that the addition of SP from good-freezer boars could improve the cryosurvival of the spermatozoa of other males [259], and even increase fertility in equines [17]. Several conclusions could be made from these experiments, namely, that spermatozoa require the presence of a certain time of exposure to SP [2,260], or even a certain proportion of SP all along (often around an empirical proportion rate of 10–20% [16,22,261], in order to maintain sperm survival and fertility post AI. The moment of SP addition varied, in most instances appearing most efficient when added post-thaw [18,22,261,262,263,264].

## 6. The Future

**Figure 8:**
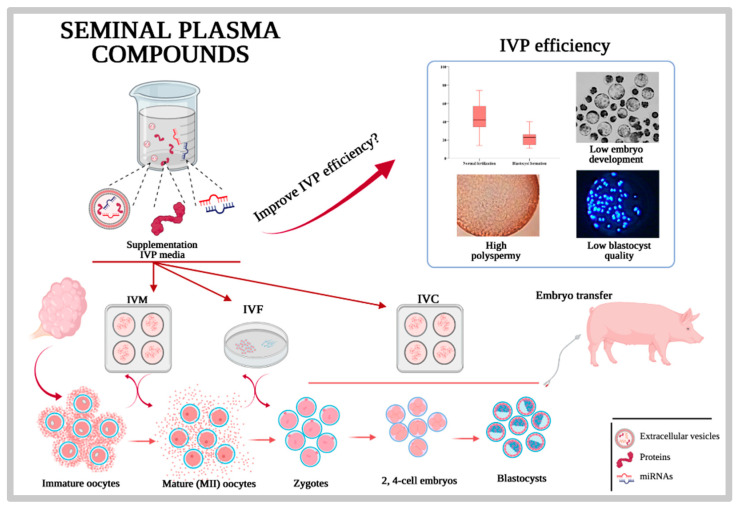
Seminal plasma can via specific proteins, native or synthetic extracellular vesicles and miRNas improve the nowadays sub-optimal in vitro porcine embryo production (IVP).

In the end, would it be at all possible to use exogenous SP components to improve fertility after AI with extended, cooled, or frozen–thawed semen? Probably, the pertaining experimentation provides us with irrefutable proof that the associations seen between diverse molecules in SP and the fertility of the males go beyond that: associations. The reason for these words of caution resides in the fact that, despite many molecules, particularly proteins or antioxidative enzymes were presented as directly related to male or semen fertility, and their effect on semen has been elusive, sometimes arguing that the relation-to-effect seen was probably concerted, multifactorial and not simply related to one particular molecule. Moreover, the administration of such molecules, and particularly its resistance to degradation and its capacity to act onto living spermatozoa, are considered relevant hurdles to be explored. Hope is now focused on the growing knowledge on EVs and their functional cargo, mostly because they would be protected by the membrane-built EVs and because they would probably be the best delivered to living spermatozoa [175]. There are several strategies, such as isolation and preservation of the EVs derived from the SP of recognized and well-documented highly fertile males, to be used as additives for improvement of fertility [265]. The rationale follows proven experiments where the SP of good freezers could improve the cryosurvival in less-good freezers [259]. However, one matter is cryosurvival and another fertility as such, although they are highly related. Another approach could be the design of “synthetic EVs”, mimicking liposomes [266], which have been proven as relevant for protecting spermatozoa during cooling and post-AI. An intermediate approach is to combine the isolation of native SP EVs with techniques to upload specific molecules, relevant for fertility. All these approaches are technically possible today, particularly when intending to improve in vitro embryo production (IPV) (Figure 8).

However, we still need to determine the mechanisms ruling fertility for many of the molecules present in these SP-EVs, and the role they play on the immunoregulation of the female genital tract, modulating the capacity of the female to allow the presence and development of pregnancy to term, and for some species of interest, particularly for their value as livestock but also as suitable animal models for human reproduction.

## 7. Conclusions

This review has critically summarized the current knowledge of the roles the SP and its particular components apparently at play in ruling fertility and prolificacy. Its focus has been to provide a comparative view, including humans, and considering suitable experimental animal models and livestock as well. The SP’s importance was particularly discussed in relation to sperm survival, function and signaling to the female immune system towards fertility modulation. Considering many in vivo findings with major relevance for the development of diagnostic/prognostic biomarkers and the refinement of reproductive strategies, particular interest has been put on the immunoregulation of the female genitalia, since it appears determinant for fertility in all species and even animal classes explored so far. Based on the available knowledge, it is expected that novel experimental research on SP EVs shall be of utmost relevance to advance not only our knowledge but our capacity to manipulate fertility.
